# A text-based conversational agent for asthma support: Mixed-methods feasibility study

**DOI:** 10.1177/20552076241258276

**Published:** 2024-06-17

**Authors:** Darren Cook, Dorian Peters, Laura Moradbakhti, Ting Su, Marco Da Re, Bjorn W. Schuller, Jennifer Quint, Ernie Wong, Rafael A. Calvo

**Affiliations:** 1 4615 Dyson School of Design Engineering, Imperial College London, London, UK; 2 Imperial College Healthcare NHS Trust, London, UK

**Keywords:** Digital health, ehealth, chatbots, asthma, WhatsApp, conversational agents, healthcare technology

## Abstract

**Objective:**

Millions of people in the UK have asthma, yet 70% do not access basic care, leading to the largest number of asthma-related deaths in Europe. Chatbots may extend the reach of asthma support and provide a bridge to traditional healthcare. This study evaluates ‘Brisa’, a chatbot designed to improve asthma patients’ self-assessment and self-management.

**Methods:**

We recruited 150 adults with an asthma diagnosis to test our chatbot. Participants were recruited over three waves through social media and a research recruitment platform. Eligible participants had access to ‘Brisa’ via a WhatsApp or website version for 28 days and completed entry and exit questionnaires to evaluate user experience and asthma control. Weekly symptom tracking, user interaction metrics, satisfaction measures, and qualitative feedback were utilised to evaluate the chatbot's usability and potential effectiveness, focusing on changes in asthma control and self-reported behavioural improvements.

**Results:**

74% of participants engaged with ‘Brisa’ at least once. High task completion rates were observed: asthma attack risk assessment (86%), voice recording submission (83%) and asthma control tracking (95.5%). Post use, an 8% improvement in asthma control was reported. User satisfaction surveys indicated positive feedback on helpfulness (80%), privacy (87%), trustworthiness (80%) and functionality (84%) but highlighted a need for improved conversational depth and personalisation.

**Conclusions:**

The study indicates that chatbots are effective for asthma support, demonstrated by the high usage of features like risk assessment and control tracking, as well as a statistically significant improvement in asthma control. However, lower satisfaction in conversational flexibility highlights rising expectations for chatbot fluency, influenced by advanced models like ChatGPT. Future health-focused chatbots must balance conversational capability with accuracy and safety to maintain engagement and effectiveness.

## Introduction

Asthma is a chronic respiratory condition that affects 12% of the UK population and over 300 million people worldwide.^1^ Despite being a common condition, symptoms vary widely from person to person, and the serious risks posed by asthma are often poorly understood by patients. For example, in a recent study, over 71% of patients overestimated their perception of disease control.^2^ Moreover, one in six people in the UK are unaware that asthma can be fatal.^3^ An underestimation of asthma risk likely contributes to the underutilisation of basic healthcare services,^4^ which in turn perpetuates poor asthma health literacy, creating an unfavourable loop for asthma health outcomes. Access to traditional healthcare services entails barriers, including time off work, wait times for appointments and proactive effort on the part of the patient to arrange appointments. Patients self-assessing their asthma as low risk may not consider these costs worthwhile.

Therefore, reaching people by other means, such as leveraging communication channels that patients already use daily (and which provide anywhere, anytime access), could provide a powerful way to improve risk self-assessment, health literacy and motivation to access traditional care.

Chatbots can provide easy access to information anytime with the benefit of a familiar conversational format. For this reason, the use of chatbots to supplement traditional healthcare services has received support from both patients and medical practitioners.^5–8^ Consequently, the numbers of chatbots for applications in healthcare are increasing. Their benefits have already been demonstrated in prescription adherence,^9^ talk therapies^10^ and symptom monitoring.^11^

The application of chatbots as an asthma support tool has been explored with promising results in a handful of initial studies conducted with children and adolescents.^12–14^ While these studies suggest promise for the use of chatbots for asthma care, they have been limited to small groups of paediatric patients. Yet, there is reason to believe that the opportunity for adults would be just as promising. According to recent figures, 80% of 16–64-year-olds in the UK already use WhatsApp.^15^ However, research on leveraging such widely used platforms for supporting adults with asthma is still needed and could reveal new opportunities for improving outcomes.

In this paper, we present the outcomes of a feasibility study of an asthma support chatbot, including evaluations of its usability and efficacy for improving self-management, health literacy and service access. More specifically, the chatbot was designed to provide asthma patients with:

- An assessment of their asthma attack risk within the next 3 months via a novel risk model;^16^
- A way to assess their asthma control over time via the Asthma Control Questionnaire, ACQ;^17^
- Asthma health literacy education via conversations about the disease, proper medication use, potential triggers and management strategies;
- Personalised encouragement to access basic healthcare services, such as their GP or asthma nurse, customised to their levels of risk and control;
- Access to the above features is through a chatbot interface available on WhatsApp and on a website.

The research team identified these components as promising approaches for addressing the existing asthma health challenges introduced above. Specifically, poor risk self-assessment would be improved by providing users with easy access to an evidence-based risk assessment tool; overestimation of asthma control would be addressed by providing an easy way for users to assess their control more objectively using a gold standard control measure (the Asthma Control Questionnaire, or ACQ) and utilisation of health services could be improved by including personalised encouragement to contact a GP or asthma nurse based on a user's specific responses. Furthermore, by weaving personalised information about asthma triggers medication, and management throughout the conversation, health literacy might be improved, leading to better self-management. An experimental and optional voice-based risk assessment feature was also included. Although the feature is still in the technical development stage and not at one in which it can reliably assess asthma attack risk (and therefore not a functional component of the system), we report on user engagement with the feature as an indication of user interest in voice-based approaches to risk self-assessment.

## Related work

Mobile health is a rich area of research that has included work on asthma.^18^ Chatbots for health have also demonstrated benefits across various conditions and tasks, including delivering information, providing elements of basic care and supporting behaviour change through personalised dialogue.^19,20^

More narrowly, previous work combining the two – using *mobile chatbots* for *asthma* care – includes three studies investigating conversational agents for young people with asthma. First, Rhee et al.^14^ created a smartphone-based self-management tool that identified asthma symptoms from SMS messages. This system was tested by 15 adolescents and their parents. Response rates by the young participants were high at 81%–97% for daily messages. Moreover, study findings demonstrated that participant awareness of symptoms and triggers, their sense of control and their treatment adherence increased.

In a 2019 study, Kadariya et al.^12^ presented a chatbot designed to interact via text and voice through an Android application. The chatbot, called kBot, was designed for children with asthma aged 8–15 years old. Although the study demonstrated positive technology acceptance and usability scores from clinicians (*N* = 8) and researchers (*N* = 8), no testing with users was reported.

Most recently, Kowatsch et al.^13^ described a chatbot called MAX, designed to promote asthma education and self-management skills (such as inhalation technique) for 10–15-year-olds. MAX combined three forms of communication: health professionals could email MAX, patients could access it through a mobile app and family members could communicate with it via SMS. Study results demonstrated high user acceptance and improved cognitive and behavioural outcomes.

Results of all three of the studies provide early evidence for the positive role chatbots stand to play in asthma management. However, they were limited to small groups and interventions exclusively for paediatric patients. As such, there is much space for further research, not only for further building the evidence base for paediatric support but also for investigating the unexplored area of chatbot-based asthma support for adults.

## Methods

This study received ethical approval from Imperial College London's Science, Engineering and Technology Research Ethics Committee (#21|C7403) and was pre-registered. An initial protocol paper outlined a study plan for developing and evaluating the feasibility of a chatbot designed to assist patients with asthma with risk self-assessment and self-management.^21^ The user research and co-design phase of the project, which involved respiratory specialist physicians, asthma nurses, representatives from an asthma advocacy organisation and asthma patients, is described in Moradbakhti et al.^22^

### Expert and patient participation and oversight

Our approach to ensuring safety, accuracy and relevance concerning the content created for the chatbot involved a combination of four approaches.

1. *Patient and physician mock interviews*: The conversational design of the chatbot was informed by conducting a series of mock doctor–patient appointments between the asthma specialist clinicians on the research team and asthma patients from an NHS trust patient advisory group. In these sessions, the doctor and patient were asked to conduct a typical asthma check-up session, including standard questions they would normally ask. A researcher conducting the interviews (DP) asked follow-up questions to expand on the interaction and inform the dialogues.
2. *Full review of content by specialist physicians*: Before the chatbot was shared with patients, the full tree of potential dialogue paths that could be provided by the chatbot (i.e. the full multi-pathway script) was reviewed by two physicians on the research team with specialist expertise in respiratory medicine (JQ and EW). They confirmed the accuracy and provided feedback to improve the dialogues.
3. *Chatbot testing (asthma specialists, engineers, designers)*: Before the chatbot was shared with patients, a series of prototype versions were provided iteratively to the development team, three asthma specialist nurses and a general medical practitioner (outside of the research team). This specialist asthma team tested each iteration and provided feedback on the dialogue and functionality via an online form. They drew on their extensive experience with the kinds of questions, concerns and misconceptions presented by patients with asthma. This testing phase also helped developers identify any faults or bugs in the setup before the pilot.
4. *Review of dialogues between chatbot and patients*: As an added safety measure, once the chatbot was made available to patients, transcripts of patient conversations with the chatbot were reviewed by our specialist physicians (EW and JQ). No problems were identified.

### Interview COREQ data

The mock doctor–patient interviews functioned as a role-play between a real asthma patient and a real respiratory specialist from the research team acting out a typical asthma medical appointment. The goal was to help the project designers understand the context and flow of conversation between doctor and patient with respect to asthma dialogues. Therefore, these mock interviews did not represent a traditional in-depth qualitative interview study, where a series of specific questions are asked of many participants until saturation is reached. There were just three, and they were intended to inform project development rather than produce research evidence. Nevertheless, we have included the COnsolidated criteria for REporting Qualitative research checklist (COREQ) to provide additional information, where applicable, in a standardised way. The reference checklist is included as a supplementary file, and COREQ information is provided in the following paragraph.

DP facilitated the mock doctor–patient interviews. She holds a PhD with associated training in qualitative methods and held the position of Research Fellow at the time of study. The facilitator had no relationship with the patients taking part in the interviews but was acquainted with the doctors as they were co-investigators. The facilitator has a history of doing academic research on health technology and reports no conflicts of interest. The goals of the research were explained to the patients in advance, and consent was obtained. A convenience sample of patients was used as the patients participating were part of an existing patient advisory group pertaining to the Imperial NHS Trust. Patients were invited to participate via email. Three volunteered to participate (two females and one male between the ages of 18 and 35), and none dropped out. Interviews (each lasting about 30 minutes) were conducted online via a video conferencing platform, and audio was recorded. DP performed informal thematic analysis across the transcripts to highlight themes (no themes were determined in advance) that could be helpful in informing the conversational design of the chatbot.

### Feasibility study

#### Overview

The study was conducted iteratively over three waves, each a month long. Wave 1 began in April 2023, Wave 2 in July 2023 and Wave 3 in October 2023. The time between waves was used to review and respond to feedback, add additional functionality and refine the user experience.

The focus of this study was to measure the feasibility (including usability and potential effectiveness) of our chatbot, Brisa. Here, *feasibility* refers to the practicality and ease of implementing Brisa in a real-world setting, encompassing user interest, engagement levels, resource utilisation and early indicators of improved behaviour or health outcomes. *Usability* focuses on the chatbot's user-friendliness, including its interface design, ease of navigation and user satisfaction. At the same time, *potential effectiveness* examines preliminary indicators of the chatbot's capacity to improve asthma knowledge and self-management. This was evaluated through changes in self-reported assessments of the chatbot's value and helpfulness, self-reported indicators of behaviour change and asthma control scores before and after using the chatbot.

A summary of the measures we deployed to assess the chatbot is as follows.

1. *Retention rate*: Refers to the percentage of users who used Brisa at least once compared to all users who agreed to participate.
2. *User engagement*: Shown by the number of conversations a user had with Brisa over the 28 days. We define a conversation as a sequence of at least three messages the user sends that are not broken by a period greater than 12 hours.
3. *Task completion:* Given by the percentage of users who successfully answered all questions for the risk assessment compared to those who dropped out. Risk assessment was the first dialogue initiated by the chatbot.
4. *User satisfaction*: Was gathered from questions asked during the exit survey. These questions focused on the naturalness and consistency of the chatbot's language, and the helpfulness of the information provided. We used the Net Promoter Score^23^ as our primary measure of user satisfaction.
5. *Differences in pre- and post-intervention measures of the ACQ*: Users were required to complete the ACQ as a pre-requisite to accessing the chatbot. They then completed it again after having access for 28 days. We compared the difference in the ACQ scores, where a lower score indicates greater disease control, as an initial indicator of the chatbot's potential effectiveness.
6. *Self-report behaviour change indicators*: After the chatbot suggests a particular action a user can take to improve their asthma, it asks, ‘Is this something you think you would consider doing?’ We used the percentage of positive responses as an early indicator of potential behaviour change.
7. *User perceptions of helpfulness*: During the exit questionnaire, participants were asked if they felt the chatbot helped manage their asthma and if they would recommend it to others (via the Net Promoter Score). We interpret the percentage of users who positively affirm these statements as another preliminary indicator of potential efficacy.

#### Recruitment

Our initial recruitment strategy involved screening potential participants via the survey recruitment company Prolific.^a^ Our inclusion criteria included a minimum age of 18 years, living in the UK and being diagnosed with asthma. Participants were offered a £10 gift voucher as compensation for their time.

For waves 2 and 3, the study was promoted through a series of paid and non-paid advertisements on social media sites, including Facebook, Instagram, and LinkedIn (see Appendix A for examples of our recruitment campaign). Social media recruitment increased the response rate considerably, although it also contributed to an increase in fraudulent bot responses. To counteract this, a CAPTCHA was added to the entry questionnaire as an additional verification measure.

Participants who clicked on one of the social media advertisements were prompted to complete the entry questionnaire and asked to select how they preferred to interact with Brisa (choosing from WhatsApp or via a website). Those eligible and who completed the survey were added as users by a research team member (DC or TS) and sent instructions on interacting with the chatbot. Individual users were only permitted to participate in one of the three waves.

#### Questionnaires

Participants were invited to complete an entry questionnaire hosted on Qualtrics before being given access to the chatbot for 28 days. On day 28, they were sent an exit questionnaire (also administered in Qualtrics).

1. *Entry questionnaire*: The entry questionnaire consisted of 25 multiple-choice questions and 4 open-ended free-text questions. The questions established a baseline of participants’ asthma control level using the standard Asthma Control Questionnaire (ACQ)17. The questionnaire asked about participants’ prior experience and comfort with technology and their trust in the UK health service. It also gathered informed consent, demographic details (gender, age, ethnicity and level of education attained) and their platform preference for using the chatbot (WhatsApp or web).
2. *Exit questionnaire*: The exit questionnaire consisted of 42 multiple-choice questions and 10 open-ended text questions. An identical ACQ and a series of questions relating to the experience of using the chatbot were included. These questions included the Net Promoter Score^23^ and custom questions to elicit satisfaction with the chatbot’s tone, language, consistency, features, accuracy and value. Open questions relating broadly to aspects of the user experience (e.g. ‘What did you like best about the chatbot?’) were also included.

#### Interaction data

With user consent obtained during the entry questionnaire, we collected comprehensive system-generated data from user interactions with the chatbot, including all user input (typed text and button responses), ACQ and risk model scores, symptom selections and asthma trigger selections made as part of the conversation. Data were stored and distributed across two PostgreSQL databases hosted on a Microsoft Azure cloud server. Each data point was linked to a unique user identifier (UUID) associated with each user, ensuring that the information remained anonymous and not tied to personal user details.

A reminder to interact with the chatbot was sent via WhatsApp and email each week during the 28 days, although reminders via WhatsApp were only sent to participants of Wave 3.

#### Statistical analysis

We performed statistical analyses on the entry and exit questionnaire data and the interaction data stored in our databases. Specifically, we examined the data for a statistically significant difference in user engagement based on the version of the chatbot selected by users (WhatsApp and web). We also explored differences in user engagement across the three waves. Furthermore, we sought to examine differences in asthma control before and after interacting with the chatbot to indicate Brisa’s potential efficacy as an asthma-support tool. All statistical tests used non-parametric variants due to deviation from normality and were performed using the NumPy and SciPy packages in Python (version 3.9).

### System description and functionality

**Figure 1.**
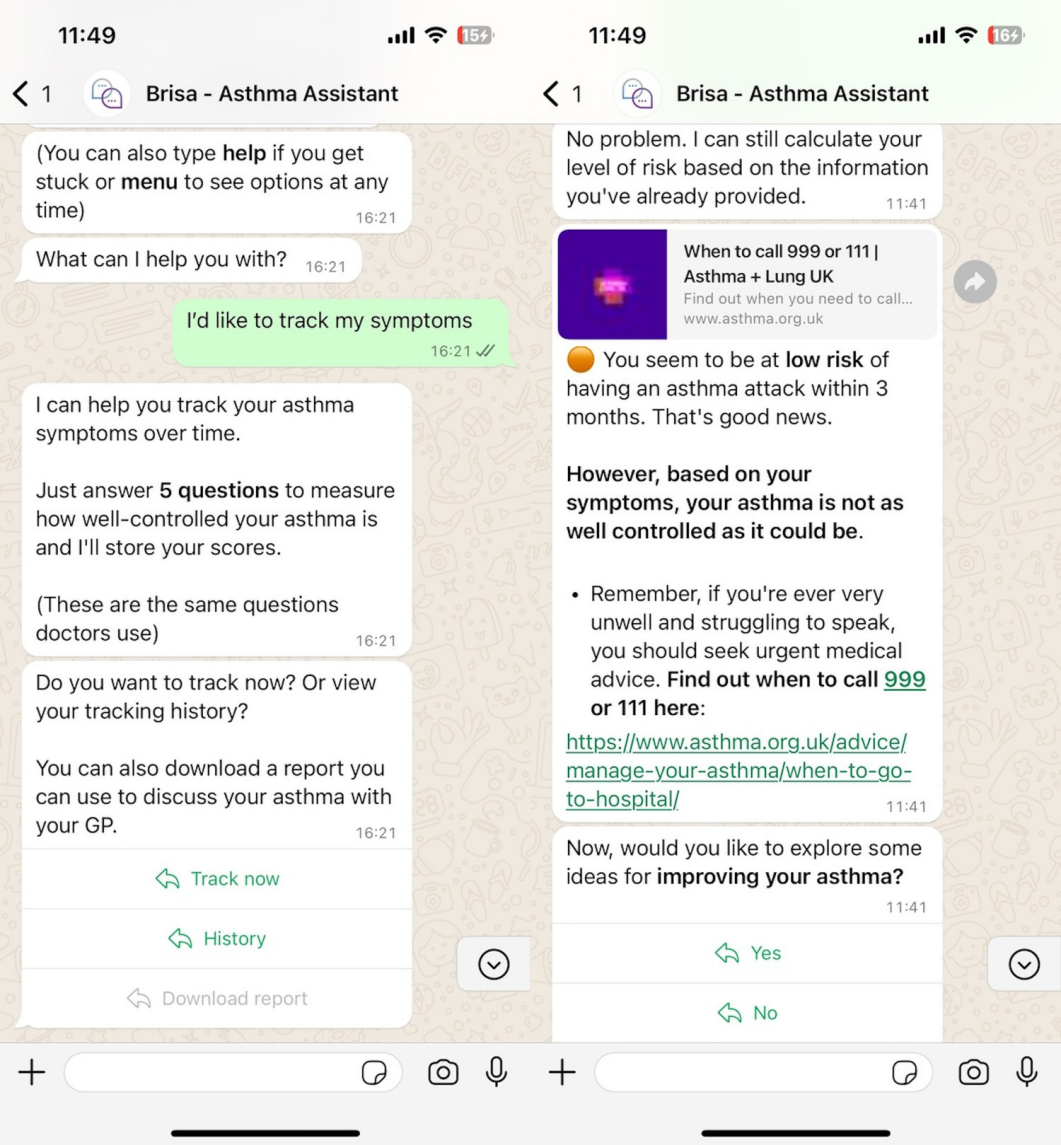
Demonstration of the Brisa dialogue during the asthma tracking function (left) and risk calculation (right) – WhatsApp version.

Our chatbot, Brisa (see Figure 1), comprises a JavaScript frontend that supports interfaces via WhatsApp and web chat and a backend FastAPI server hosted in the Cloud via Azure microservices. Conversation dialogue was designed using Voiceflow, a low-code chatbot builder. A full description of the underlying architecture is the focus of ongoing work.

Brisa was designed to assist users with a series of asthma-related tasks:

- Measure the risk of an asthma attack via the model described in Kallis et al.;^16^
- Track users’ asthma control weekly via the ACQ;^17^
- Answer questions regarding asthma management;
- Help users identify triggers that exacerbate their symptoms;
- Provide strategies to help users manage their triggers.

The following section summarises Brisa's main features.

*Authentication*: To access Brisa, users must undergo a secure authentication process. User access is linked to specific identifiers: a UK phone number for WhatsApp users or an email address for web-based users. Upon initiating a conversation with Brisa, the system employs a 32-character UUID to verify the user’s identity. This ensures that access to Brisa is restricted and secure, safeguarding research integrity and user privacy.

*Personalisation*: The UUID generated from the authentication step tracks user activity when interacting with Brisa. Brisa asks for a nickname at the start of the first interaction, which is then stored and used to create a personalised greeting when the user next interacts. Other steps to tailor the experience to the user include altering risk messaging based on whether the user is deemed to be at high or low risk of an asthma attack or based on whether they have identified specific asthma triggers.

*Risk of an asthma attack*: This feature calculates the user's risk of an asthma attack within three months. The underlying algorithm is based on the user's answers to a 19-item questionnaire drawn from a regression model by Kallis et al.^16^ The model was developed from data on 1.2 million individuals with asthma. The resulting score categorises users into high- or low-risk groups,^b^ with high-risk individuals encouraged to contact a healthcare professional as soon as possible. New users are prompted to use this feature when first conversing with the chatbot.

*Asthma tracking*: Users are provided with the option to track their asthma weekly. The chatbot asks questions based on the asthma control questionnaire (ACQ).^17^ Specifically, we use ACQ-5, a gold-standard five-item questionnaire that measures the level of asthma control based on the severity of symptoms on sleeping, waking, daily activities, breathing and wheezing. Users score themselves on a 7-point Likert scale of increasing severity for each item. The ACQ is designed to measure the level of asthma control but lacks an established threshold for controlled and uncontrolled asthma.^24^ After consultation with medical professionals, we interpreted scores above 4 as ‘uncontrolled’, between 2 and 4 as ‘partially controlled’, and under 2 as ‘well controlled’. We averaged scores across the five items to give an overall indicator and then tailored messaging accordingly. Users were encouraged to track their symptoms weekly via notifications sent directly from Brisa on WhatsApp^c^ or as an email for web users. In addition, Brisa stored results so users who had tracked their symptoms more than twice could review their tracking history.

*Identify triggers*: Users were invited to engage with a series of questions to pinpoint potential asthma triggers. The chatbot specifically probes for four prevalent trigger types: pets, cold weather, seasonal allergies and air pollutants. Although asthma triggers are numerous, and a final chatbot version would need to include dialogues for many more, these four were selected as an initial proof-of-concept set for the feasibility study. Upon completing these questions, users receive a summary highlighting their relevant triggers and are invited to receive further information on managing each identified trigger.

*Improve symptoms*: Brisa provides users with guidance and information about managing common asthma symptoms, verified for accuracy by medical professionals. To personalise this support, the chatbot asks users an open-ended question about their symptom triggers. Responses are processed using generative artificial intelligence (GAI; GPT-3.5-turbo), identifying key entities. These entities are then mapped to a list of predefined variables, ensuring a controlled interaction with the GAI and preventing direct user exposure to the generative model. If a user's response does not align with these variables, Brisa offers default options to assist them further.

*Generate a report*: Users can request a comprehensive summary of their interactions with Brisa. This report includes their risk level, symptom-tracking history and any identified triggers. It is designed to be a conversational aid for discussions with healthcare professionals. To create a report, users must have completed at least two symptom-tracking sessions and undergone a risk assessment for an asthma attack. The report is securely stored in the Cloud. To ensure privacy, users receive a temporary link to download it, and the report excludes any personally identifiable information.

*Ask a question*: This feature enables users to inquire about asthma-related topics beyond the predefined dialogue options in the menu provided by Brisa. When a user poses a question, it is first cross-referenced with a database containing question–answer pairs verified by medical professionals. Brisa provides the corresponding approved response if the user's question aligns with one in the database. For questions not covered in the database, GPT-3.5-turbo automatically generates an answer that is not sent to the user but stored for review in a backend system. These generated responses later undergo periodic review by medical professionals for accuracy and relevance. Once validated, they are incorporated into the database, enriching the resource for future user queries.

## Results

### Demographics

We recruited a total of 150 members of public with asthma to test Brisa across the three waves. Wave 1 comprised 12 users (recruited via Prolific – a research participation platform), 38 users for Wave 2 and 100 users for Wave 3 (the last two groups were recruited via social media advertising).

Sociodemographic statistics across waves were collected during the entry questionnaire and are reported in Table 1. The age of participants was consistent across the three waves, with an average of 39.36 years (*SD *= 12.57 years).

**Table 1.** Participant sociodemographic information per wave and combined.

Sociodemographic variables		Wave 1	Wave 2	Wave 3	Total
Total users	–	12	38	100	150
Age (years)	Mean	40.42	38.47	39.57	39.36
	SD	10.94	16.94	12.16	12.57
Gender	Male	7 (58%)	10 (26%)	24 (24%)	41 (27%)
	Female	5 (42%)	28 (74%)	74 (74%)	107 (71%)
	Non-binary	-	-	2 (2%)	2 (1%)
Education	Secondary school	2 (17%)	4 (11%)	12 (12%)	18 (12%)
	Upper secondary (UK sixth form)	4 (33%)	16 (42%)	25 (25%)	45 (30%)
	Undergraduate	4 (33%)	11 (29%)	34 (34%)	49 (33%)
	Postgraduate	2 (17%)	6 (16%)	20 (20%)	28 (19%)
	Other	-	1 (3%)	9 (9%)	10 (7%)
Minority ethnic group	Yes	2 (17%)	5 (13%)	16 (16%)	23 (15%)
	No	10 (83%)	33 (87%)	84 (84%)	127 (85%)
Platform	WhatsApp	9 (75%)	32 (84%)	83 (83%)	124 (83%)
	Web browser	3 (25%)	6 (16%)	17 (17%)	26 (17%)

The gender split in Wave 2 and Wave 3 was similar; most participants (74%) identified as female, with males more common (58%) in Wave 1. Two individuals identified as non-binary during Wave 3.

For education level, most participants (82%) completed secondary education (high school) at a minimum. In total, 15% identified as part of a minority ethnic group. Lastly, the WhatsApp version of the chatbot was consistently the most preferred version of Brisa, with 83% of all users electing to use WhatsApp over the web browser version.

### Expert review data

We collected and analysed 54 feedback items from our review group during the expert review phase of our study. Feedback was categorised into different types based on the nature of the issues or suggestions.

1. *Dialogue issues*: Comprised 13% of the feedback, focusing on problems related to the chatbot's conversational design (e.g. awkward phrasing). These issues were resolved through consultation among the research team.
2. *Intent/Response recognition*: Accounted for 5.6%, highlighting issues where the chatbot failed to understand or respond to user inputs correctly. These were addressed by improving the Natural Language Understanding model in Voiceflow.
3. *Conversational dead ends*: Formed the largest category at 37%, indicating instances where conversations with the chatbot reached an impasse with no clear continuation path. These issues were resolved by creating a default dialogue path where the chatbot apologised and explained it could not assist before providing alternative options.
4. *Typos and minor errors*: Spelling and grammatical errors in the chatbot's responses comprised 5.6% of the feedback and were corrected by the research team.
5. *Ideas for improvement*: Constituted 22.2% of the responses, offering constructive suggestions for enhancing the chatbot's functionality and user experience.

The remaining 17% of the feedback did not fit these predefined categories. These issues consisted of minor software-related bugs and errors that were resolved by the developer team.

Based on these insights, we implemented a series of refinements to the chatbot, addressing the identified issues and incorporating the suggested improvements. These modifications were essential in preparing the chatbot for use by patients in the subsequent user testing phase of the study.

### Iterative results and development summary

Brisa underwent three iterations, starting with a smaller test group and expanding the sample as the prototype improved with user feedback. Results and feedback gathered from participants via the exit questionnaires were reviewed by the research team and used to inform bug fixes, improvements and feature development for the next wave. An overview of the changes made during each wave is described below.

#### Wave 1

The expert review phase informed the initial development of Wave 1. The available functionality included the asthma attack risk assessment, asthma control tracker, trigger identification dialogue and symptom improvement dialogue. Weekly email notifications were sent to users (WhatsApp and web users).

After interacting with Brisa, 92% (11/12) of users completed the exit survey. Participants highlighted the symptom tracker as the feature they liked best, with the risk calculator and ease of use also regarded highly. Participants expressed dissatisfaction with the linearity of the dialogue, remarking that it took more work to exit a particular dialogue path once it had started. Several users expressed frustration when Brisa failed to recognise a response. When asked how Brisa could be improved, participants suggested receiving notifications and reminders directly from the chatbot and making the dialogue more sophisticated and able to handle unexpected user queries.

#### Wave 2

Wave 2 made several refinements to Wave 1. First, we changed our recruitment strategy from a single advertisement via Prolific to a multi-advertisement campaign on social media. As part of this effort, we created 5 advertisements with small variations in the messaging. This campaign successfully increased the number of users by over 200% to 38 during the three-week recruitment period.

In summary, we implemented the following updates for Wave 2:

- *Frequently asked questions (FAQs)*: a feature allowing users to ask free-form questions and receive answers that had not been explicitly defined within the dialogue;
- *Voice recording for the asthma control tracker*: duplicating the functionality already deployed in the risk calculator but also providing it as part of the tracker;
- *View user history*: enabling users to see all prior tracking (ACQ) scores;
- *Further information*: An enhanced explanation of the Kallis et al.^16^ risk model was used to predict the likelihood of an asthma attack, including a link to a website where a user could access further information.

Despite a substantial increase in users compared to Wave 1, only 45% (17/38) of Wave 2 users completed the exit survey. Of these, just under half (47%, 8/17) indicated they would likely recommend Brisa to another person with asthma (via the Net Promoter Score). Users were impressed by Brisa's level of personalisation, human-like qualities and the amount of information provided. By comparison, 24% (4/17) were uncertain if they would recommend Brisa and 29% said they would not. These users were dissatisfied with the amount of question repetition and expressed a desire for more variety in the chatbot's responses and knowledge base. Suggestions for improvements included the opportunity to speak to a human if Brisa did not understand a particular input.

#### Wave 3

In response to feedback from Wave 2, we implemented the following additions for Wave 3:

1. *WhatsApp reminders*: WhatsApp users could receive reminders directly from Brisa in WhatsApp rather than via email.
2. *Help menu*: A globally accessible ‘Help’ menu was added that could be used to exit dialogue paths if the users became stuck.
3. *Links to live help*: While we could not offer the ability to talk to a human in-house, in response to this user feedback, we did include a link to the Asthma & Lung UK WhatsApp contact number, which provides access to a live asthma nurse during opening hours.
4. *More FAQs*: We increased the number of approved answers in our FAQ database.
5. *Visual tracking history*: A user's tracking history could be viewed across a graphical timeline rather than just via text.
6. *History report*: A reporting feature that summarises a user's tracking history and risk scores that can be downloaded as a PDF file and shared with a doctor (see Appendix C for an example).

The recruitment campaign for Wave 3 was identical to that in Wave 2, except that we increased the number of advertisements placed on Facebook, LinkedIn and Instagram. This resulted in 100 users for Wave 3, which we reached in 21 days. 52% (52/100) of Wave 3 users completed the exit survey. Of these, only 27% (14/52) said they would recommend Brisa to a friend with asthma, showing a decline in user satisfaction since Wave 2.

Users noted a need for more personalisation, repetitive messaging and robotic dialogue. A minority of users preferred a more conventional app instead of interacting via WhatsApp. However, when asked in open text about the features they liked most, users rated the ease of access, 24/7 availability and the quality of the information provided.

### User retention, satisfaction and engagement

#### User retention

User retention was measured as the percentage of respondents who, having filled out the entry questionnaire, went on to access the chatbot at least once during the 28 days. The total retention rate across the study was 74% (111/150). Between the individual waves, Wave 1 garnered the highest retention rate at 92% (11/12), with Wave 2 generating 61% (23/38) and Wave 3 receiving 77% (77/100).

#### Engagement

Engagement was measured by the number of conversations an active user^d^ had with Brisa, where a higher number of conversations indicates greater engagement. Throughout the study, the average active user engaged with the chatbot twice (2.38 conversations, *SD* = 1.51) and sent 24.74 (*SD* = 18.91) messages per conversation. Wave 1 users averaged 4.2 (*SD* = 1.99) conversations and sent an average of 18.36 (*SD* = 15.79) messages per conversation. Wave 2 users averaged 1.77 (*SD* = 0.92) conversations and sent 27.18 (*SD* = 18.97) messages to Brisa. Users in Wave 3 averaged 2.32 (*SD* = 1.41) conversations with Brisa, sending 25.73 (*SD* = 19.34) messages per conversation. The maximum number of conversations by a single user throughout the study was 7 (in Wave 3).

Table 2 splits each wave into subgroups based on whether they were WhatsApp or web users. Overall, the number of conversations was higher for WhatsApp users (average = 2.46) compared to web users (1.87). A Mann–Whitney U test revealed a non-significant difference in user engagement between the two platforms (p = 0.15).

**Table 2.** Active user engagement (average (+/– SD) conversations) split by the platform used to access the chatbot. Higher values indicate greater user engagement**.**

Platform	Wave 1 (n = 11)	Wave 2 (n = 23)	Wave 3 (n = 77)	Total (n = 111)
WhatsApp	4.86 (1.87)	1.85 (0.93)	2.39 (1.45)	2.46 (1.55)
Web	2.67 (1.53)	1.00 (0.0)	1.8 (1.03)	1.87 (1.13)

A Kruskal–Wallis H test was then used to compare user engagement between the three waves. We observed a statistically significant difference in user engagement (*H* = 12.63, p < .01) when adjusted for multiple comparisons,^e^ with a medium effect size (*η*^2^ = 0.08). Dunn's test was used as a post-hoc measure of the pairwise differences, revealing significantly higher engagement for Wave 1 over Waves 2 and 3 (p < .05). In contrast, the differences in user engagement between Waves 2 and 3 did not approach significance.

### Task completion

#### Asthma attack risk assessment

Task completion was based on the proportion of users who answered all risk assessment questions compared to those who only started the dialogue. Understanding the likelihood of an asthma attack was suggested to users as a first activity during their initial conversation with Brisa. Overall, 86% (95/111) of active users started the risk calculation task during their initial interaction with Brisa. Of these, 90% (86/95) answered all risk-based questions. Furthermore, 6% (6/95) of these users completed the risk-based questions for a second time during a later conversation without prompting from Brisa.

#### Voice recording

WhatsApp users were also invited to submit a voice recording that would be used to support research to develop a separate voice-based risk model. Web-based users could not participate in this step and were not invited. Users could easily skip this optional step. In total, 83% (66/80) of WhatsApp users invited to submit a voice recording agreed to do so.

#### Asthma control tracking

Users were also encouraged to complete the ACQ weekly through Brisa to track their asthma symptoms and symptom control over time. 95.5% (106/111) of active users completed the tracking activity at least once within 28 days. Of these, 37.74% (40/106) tracked once, 26.42% (28/106) tracked twice, 16.98% (18/106) tracked three times and 8.49% (9/106) tracked four or more times.

#### User satisfaction

User satisfaction was assessed from 69 users in Waves 2 and 3 who completed the exit survey.^f^ Questions were answered on a 5-point Likert scale, where 1 indicated strong disagreement and 5 indicated strong agreement. The percentage of users who were satisfied with Brisa was based on those with scores of 4 and above (agree or strongly agree) for each statement on the Likert scale.^g^ Most respondents agreed the chatbot was helpful (80%), private and secure (87%), trustworthy (80%), enjoyable (68%) and bug free (84%). However, a demand for greater conversational scope and fluency was reflected in lower scores for the statements: ‘the conversation was two-way’ (49%), ‘the conversation felt personalised’ (46%) and that Brisa ‘provided enough information’ (48%).

Table 3 provides an overview of how responders evaluated Brisa on all assessed aspects, such as conversational tone, helpfulness, ease of use, personalisation and overall satisfaction. We did not observe any significant difference in scores between waves.

**Table 3.** Percentage of users who scored 4 or 5 (indicating agreement or strong agreement) per item in the exit survey. Averages falling below half are bolded.

Statement	Wave 2 (n = 17)	Wave 3 (n = 52)	Total (n = 69)
The language and tone of the chatbot was appropriate.	94%	94%	94%
The conversation felt natural.	71%	60%	62%
The chatbot responded in a consistent manner.	88%	87%	87%
The chatbot gave me options that were easy to follow.	94%	83%	86%
It was easy to understand what I could do with the chatbot, and how I should do it.	82%	85%	84%
I had enough control over the conversation.	59%	62%	61%
The conversation had no bugs or abrupt endings.	88%	83%	84%
It was easy to get back on track if I made the wrong choice.	76%	75%	75%
It was easy to return to the conversation if I was interrupted.	82%	83%	83%
The chatbot's responses were helpful.	82%	79%	80%
The chatbot remembered things I told it.	76%	62%	65%
The chatbot responded in a way that felt personal to me.	53%	44%	46%
I could trust the accuracy of the chatbot's responses.	82%	79%	80%
I felt my conversations were private and secure.	82%	88%	87%
I felt I could communicate in the way I wanted to with the chatbot.	59%	58%	58%
The chatbot made me feel confident in managing my asthma.	47%	60%	57%
When using the chatbot, I felt less alone in managing my asthma.	53%	54%	54%
**When I used the chatbot, I felt the interaction went both ways.**	**59%**	**46%**	**49%**
Using the chatbot was beneficial.	76%	54%	59%
After using the chatbot, I know more about asthma.	59%	50%	52%
**I learned new things from using the chatbot.**	**47%**	**48%**	**48%**
I enjoyed using the chatbot.	82%	63%	68%

The combined Net Promoter Score (NPS) for this group of users was −7 (the full scale goes from −100 to +100), indicating a slightly negative overall satisfaction.^h^

#### Platform preference

Participants overwhelmingly opted to use the WhatsApp version of the chatbot (83% of active users), which mirrors our previous research exploring user platform preferences when interacting with a conversational agent.^22^

#### Technical reliability

We encountered minimal technical problems throughout the pilot study. During Wave 3, one web user could not access the chatbot despite following the steps provided by the research team and dropped out. Aside from this issue, no other access issues or outages were observed.

### Potential efficacy: impact on behaviour and health outcomes

#### Asthma control scores

**Figure 2.**
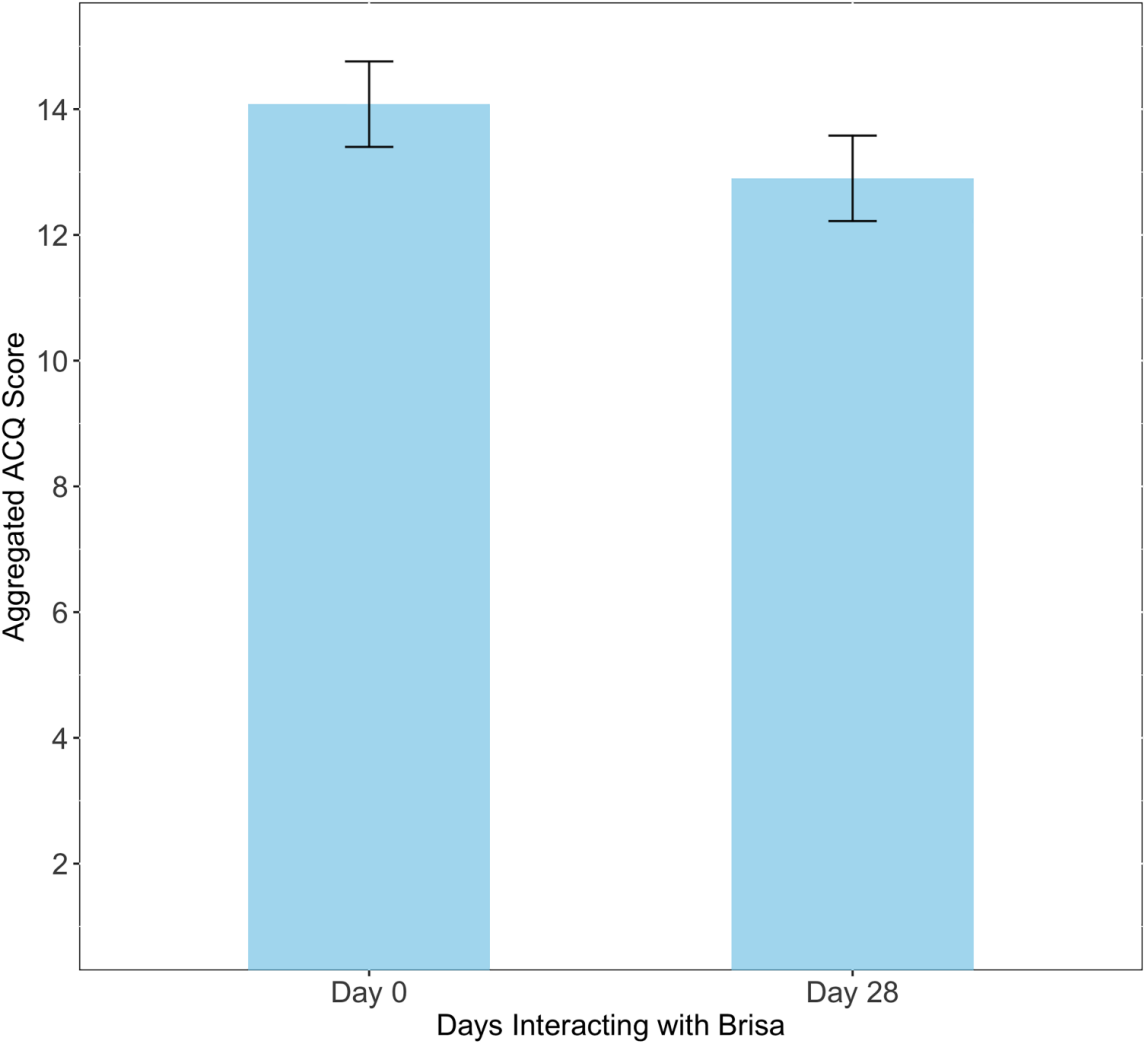
Average aggregated ACQ (+/– SE) at day 0 (before interacting with Brisa) and after 28 days. Lower scores indicate better asthma control.

One indicator for the potential effectiveness of the chatbot was measured by performing a paired comparison between the aggregated ACQ scores before and after interacting with Brisa for 28 days. Figure 2 shows the average aggregated ACQ of these two conditions. Responses across the three waves were combined to ensure adequate statistical power. Average aggregated ACQ scores on day 0 were higher (*M* = 14.08, *SD* = 5.46), indicating poorer control than the same scores on day 28 (*M* = 12.9, *SD* = 5.83). The Wilcoxon signed-rank test was preferred to a paired *t*-test after inspecting a QQ plot for indications of non-normality.

We observed a statistically significant difference in aggregated ACQ after users had interacted with Brisa for 28 days (*Z* = 691.5, *p* < .001). On average, users rated their asthma control as 8.38% better (indicated by a lower average aggregated ACQ score) after interacting with Brisa compared to that on day 0. Rank biserial correlation was used to estimate the effect size. A medium effect size (−0.33) was observed between the conditions, suggesting that interaction with Brisa correlates with improvements in asthma control. Moreover, the average improvement in asthma control after interacting with the chatbot (1.18) exceeds the Minimum Clinically Important Difference (MCID) defined in Juniper et al.^25^ indicating that the improvement is also clinically significant.

#### Behaviour change indicators

Self-assessment indicators were used to determine whether Brisa was likely to be effective in encouraging users to adopt new strategies for managing their asthma triggers. Users could identify and receive advice on multiple triggers, if applicable. Based on the interaction data, 78.38% (87/111) of active users discussed their triggers with Brisa; 68 users remarked that the weather triggered their asthma symptoms, seasonal allergies impacted 36 users and 21 users were affected by pets.

Depending on the options selected when discussing these triggers, Brisa provided suggestions on improving symptom management. For example, Brisa suggested the user cover their mouth with a scarf during cold weather if cold weather was identified as a trigger. Following this, users were asked if the suggested behavioural advice was something they would consider doing in future. Table 4 shows the response rate for each suggestion.

**Table 4.** Distribution of responses to the four behaviour change indicators. Indicators would only be presented to users if they had identified the underlying trigger (i.e., pet hair) as impacting their symptoms**.**

Behaviour change indicator	Yes	No	Not sure / I already do this
Wearing a scarf during cold weather	45.59% (31/68)	13.24% (9/68)	41.18% (28/68)
Replacing carpet with hard flooring	30.77% (4/13)	38.46% (5/13)	30.77% (4/13)
Keeping pets out of the bedroom	33.33% (1/3)	66.67% (2/3)	0.0% (0/3)
Check pollen forecast	66.67% (8/12)	8.33% (1/12)	25% (3/12)

Many users agreed they would likely wear a scarf to mitigate symptoms due to cold weather exposure (45.59% of users asked), indicating the advice was useful and new to them. Most users (66.67%) who received advice to check the pollen forecast to minimise symptoms related to hay fever agreed they would try it. Comparatively fewer users were willing to keep pets out of their bedroom (33.33%) or replace carpet with hard flooring to prevent the spread of dust and pet hair (30.77% of users).

#### Ask a question

The ‘Ask a Question’ section introduced during Waves 2 and 3 was accessed by 12% (12/100) of the active users for these waves. This is a small percentage, but it is important to note that the chatbot was not originally intended to include such a section and therefore, the recruitment text focused on Brisa’s ability to assess asthma severity and the likelihood of having an attack. As such, the sample would represent those most interested in this feature rather than those looking for an open question-and-answer feature.

Users asked 24 questions (a full list of the questions to Brisa, including 30 additional questions obtained from our specialists, is included in Appendix B). These questions are grouped around the following themes.

1. *Medication information*: Users asked about asthma medications such as Ventolin, Fostair and Montelukast. Questions focused on how the medications work, their timings and their side effects.
2. *Asthma management*: Questions were asked about asthma management strategies that had yet to be described within the existing dialogue paths. Examples include ‘How do I deal with an asthma attack?’ and ‘Can I increase my Fostair dose?’
3. *Understanding asthma*: Users asked basic questions about asthma, such as ‘Why do I use an inhaler?’ and ‘What is considered an asthma attack?’
4. *Asthma in specific contexts*: Users also asked questions about asthma in specific contexts, such as ‘Can I use an inhaler if I’m pregnant?’ and ‘Asthma in the workplace’.
5. *Symptom concerns*: Questions were asked about specific symptoms, including concerns about chest infections and ‘Why do I cough up so much phlegm?’
6. *Severity and types of asthma*: Users queried different types of asthma, such as ‘Brittle asthma’.

## Discussion

The three-wave iterative development and feasibility testing process described herein revealed numerous insights into the kinds of barriers, affordances and value propositions a chatbot can offer to deliver aspects of basic asthma care. We also believe that this value proposition was significantly impacted by the proliferation of AI generative chatbots (e.g. ChatGPT) halfway through the project, which radically shifted expectations around what an automated conversational experience should be. In this section, we synthesise study findings and reflect on using large language models (LLMs) for healthcare, including how these changes in user expectations might be balanced with safety and accuracy.

### Importance of effective expert/patient co-design, oversight and review

Our multi-pronged approach to ensuring the safety and accuracy of the chatbot during patient interactions involved a combination of approaches, including engaging with patients, specialist physicians and asthma nurses throughout the project.

The expert review methods were critical not only to ensuring safety, relevance to patients and medical accuracy but also to identifying the unique value that a chatbot can offer. For example, during mock interviews, doctors reflected on the aspects of basic asthma care and literacy for which they seldom have time during a typical appointment slot. Providing patients with accurate information about asthma and how the medication works can improve treatment adherence and health outcomes. Still, there is rarely enough time to dedicate to this during appointments that are taken up with reviewing medication and symptoms. As such, physicians and patients told the development team that the chatbot was particularly well placed to fill this gap: to answer questions and provide standardised asthma literacy information that doctors do not have time to give. This insight informed the content that was eventually included in the chatbot dialogues.

From a technical perspective, the importance and success of the expert review stage was evidenced by the relatively large amount of feedback (37%) relating to conversational dead-ends during the review phase, followed by the absence of dead-ends during the feasibility phase (see Table 3).

### Demographics

Our user sample appeared to represent a broad population, particularly for the second and third waves. While most of our users were female, reflecting the fact that Facebook advertising reached more female users, it is also true that more women develop asthma.^26^ Moreover, the observed gender split during Waves 2 and 3 was similar to that found in other health-chatbot studies.^27^ However, the reach-to-click ratio was higher for males. That is, the men receiving an ad were likelier to click on it. Compared to females, men with asthma are less likely to have a management plan, see a clinician or have access to an asthma management tool,^28,29^ which suggests an asthma chatbot could be particularly valuable for men. Future studies could investigate gender differences regarding the need and interest of using a conversational agent for asthma support.

### Findings

*Most patients interacted with the chatbot*: The final version of Brisa (Wave 3) had a retention rate of 77%, which improved on Wave 2, although it was not as high as Wave 1. We believe the drop after Wave 1 was due to a change in recruitment from Prolific to Facebook. While recruitment via paid social media advertising is often highly successful, previous studies focussing on health-related research have shown difficulties with retention rates for participants recruited through Facebook.^30–32^ It is also possible that rapidly rising expectations due to the user experience of chatbots between the first and last two waves may have contributed.

*More users opted for the WhatsApp version over the web version*: The majority (83%) of users elected to use the WhatsApp version of Brisa over the web browser version**,** aligning with our previous work indicating that users prefer to use a familiar everyday messaging platform.^22^

*Most users found the chatbot helpful*: Based on responders to our exit survey, most users agreed with statements that the chatbot was helpful (80%), private and secure (87%), trustworthy (80%), enjoyable (68%) and bug free (84%).

*Most users scored better on asthma control after 28 days of using the chatbot***:** On average, users who completed the exit survey tended to score better on asthma control after using Brisa for 28 days than before using the chatbot. This provides some evidence for the value in further investigation, for example, via a randomised control trial (RCT).

*Reminders prompted engagement*: User engagement (average number of conversations per user) increased 138% between Waves 2 and 3. This was likely due to a greater focus on sending reminders to users via WhatsApp during Wave 3 (a feature suggested in user feedback).

*Results demonstrate an interest in risk self-assessment and asthma control tracking features*: Most users (90%) completed the risk assessment, a central feature of the chatbot. In addition, most (95.5%) completed the tracking activity at least once within the 28 days. These results indicate strong evidence for users’ interest in risk self-assessment and tracking over time. Most participants completed the risk assessment only once, which is appropriate for this questionnaire, as it considers features unlikely to change over a short time and predicts the likelihood of an attack over the following 3 months.^16^ Therefore, there is no benefit to retaking the risk assessment more than once a month. For this reason, the ACQ was used for the ‘tracking’ section, as it is based on symptomatology, which can change on a weekly basis and, therefore, allows users to monitor change over time more regularly. These different use patterns are reflected in the higher numbers for repeat engagement with the tracking feature.

*Results demonstrate an interest in voice-based asthma assessment*: A large majority of users (83%) invited to submit a voice recording agreed to do so, even though it was an optional step that could easily be skipped. This suggests significant interest in a technology that could assess asthma risk or severity using the sound of one's voice. Moreover, several users suggested integration with voice assistants such as Alexa or Siri as offering a potential improvement over a predominantly text-based system.

*Users found the chatbot less conversationally fluent and knowledgeable than desired*: A demand for greater conversational scope and fluency was reflected in lower scores for agreement that the conversation ‘went both ways’ (49%), felt ‘personal to me’ (46%) and provided enough new information (i.e., ‘I learned new things from the chatbot’) (48%). Perhaps notable is that the scores on fluency went down over time rather than up, even as no changes were made to this part of the chatbot. Specifically, agreement that the conversation ‘went both ways’ dropped from 53% in Wave 2 to 44% in Wave 3, and agreement that Brisa felt ‘personal to me’ decreased from 59% to 46%. Other measures remained constant across waves. Below, we posit an explanation for the low scores in conversational fluency and why this may have worsened rather than improved across the development of the tool.

The somewhat ambivalent findings of high user satisfaction alongside low recommendation scores indicate that chatbots can indeed be effective for asthma support but that work needs to be done to meet user expectations around the experience of automated conversation. While efficacy is demonstrated by high usage and ratings of features like risk assessment and control tracking, as well as a statistically significant improvement in asthma control, lower scores for satisfaction with respect to conversational flexibility highlight rising expectations for chatbot fluency that likely produced the lower recommendation scores. We believe these results can be at least partially explained by the rising influence of advanced language models, as described below.

#### Study results in the context of the LLM explosion

During Brisa’s development (January–December 2023), ChatGPT and similar AI Chatbot technologies went from headline-making oddities to mainstream tools. At the time of writing, educators, writers and healthcare professionals continue to grapple with the disruptive changes across sectors that emerged throughout 2023.

When the research team received approval to run the present study in 2022, a pre-scripted tree-based conversational dialogue system based on conversational design best practices provided a well-established approach to creating a chatbot. No non-scripted solution existed that could be safe or smart enough to have an effective health conversation. By March 2023, ChatGPT could pass the MCAT exam.^33^

Our study was conducted iteratively over three testing waves (beginning April, July and October 2023). At the start of our study, most Americans had heard of ChatGPT but had never tried it.^34^ By the time Wave 3 had commenced, one-third of the UK adult population had used generative AI,^35^ as well as 80% of teenagers, who are using generative AI flexibly to perform various everyday tasks.^36^ A parallel study including people up to 25 years old in the UK showed that just over half (53%) had used an AI Chatbot to help them with school, email or their job and included the following participant quotation: ‘I have also used it for specific health information before seeing a health professional’.^37^

It seems reasonable to suspect that this radical leap in chatbot capabilities influenced the expectations of our study participants such that users in April would have approached our chatbot differently from users in November. We feel this is one reason for the drop in user satisfaction after Wave 1. In some sense, the results of our study inadvertently provide a record of a rapid change in user expectations about what a chatbot should be able to do and how fluently it should be able to do it. Now, in a world where it is possible to get a sophisticated response from an AI Chatbot on nearly any question, a constrained pre-scripted conversation tree feels increasingly limited and rudimentary.

However, current LLMs alone are still not safe for medical use. They continue to fabricate information, a concept known as *hallucinating*. They can inadvertently provide false information with fake references to fake sources^38^ and even falsify entire databases of fake medical data.^39^ Although their fallibility is becoming harder for the average person to detect, the problem is no less real. Therefore, the critical question is how we achieve near LLM-level fluency without introducing risk. We share reflections on this topic below, as it will likely become a prevalent issue for all conversational health technology research.

#### The safety versus fluency dilemma

In August 2023, anticipating user demand for more natural dialogue and the availability of LLM application programming interfaces, the research team began to explore whether the latter could solve the former without risking patient harm. We decided to experiment with options as part of a new FAQ section, which could be added to the chatbot and managed as a discrete area within the system that did not interfere with our primary scripted conversation space.

In this context, we explored whether ChatGPT could generate answers to questions for which the chatbot did not already have a pre-scripted answer. To constrain the model to credible sources, we directed ChatGPT to generate responses that drew on the content of specified websites, specifically, the UK's National Health Service (NHS) website and the Asthma and Lung UK website. However, specifying source content this way does not apply strict controls to what an LLM generates, as its accuracy is still fundamentally dependent on its original training data. As such, content free from hallucinations can never be guaranteed. For this reason, we were unwilling to send automatically generated responses directly to the user without expert review.

Moreover, experimenting with this approach, we observed that the *precise wording* of medical information can be important. For example, the standard advice on managing an asthma attack has been worded by experts with extreme care and refined over time to be as clear, concise, accurate and safe as possible. This carefully refined text is consistently replicated across emergency guidance and action plans. Rephrasing carefully crafted medical advice can be counterproductive, but LLMs work by paraphrasing existing information, not repeating it verbatim. Although the ChatGPT-generated answers to test questions we provided about asthma were impressive in that they felt like a natural conversation and included useful information, they were sometimes incomplete (i.e. they were not incorrect but did not always include everything a medical professional would consider important to include). For example, giving part of a good answer for how to manage an asthma attack – paraphrasing the main steps but leaving out important caveats relevant to certain medications – could still cause harm. This point may seem subtle and unimportant for some domains, yet it is of utmost significance for many high-risk healthcare situations. To demonstrate this point, Appendix D compares the NHS-approved text on what to do for an asthma attack to three responses to the question ‘What should I do if I’m having an asthma attack?’ from three LLMs (ChatGPT, Claude and CoPilot). Guiffre, You and Shung^40^ also make a similar argument from *Hepatology*.

Finally, new examples of hallucinations generated by ChatGPT and other LLMs continue to be revealed. Critically, it is important to recall that even a very low incidence of inaccurate information generated by LLMs can be harmful (if not fatal) within a health context. Therefore, to ‘first, do no harm’, we determined that the unsupervised use of AI-generated text was not an option. As such, we determined that when users typed in a question that could not be matched with an existing answer in the database, they would be directed to a reputable website and asthma helpline. In the backend, the question would trigger a generated answer via GPT-3.5 that would be sent to a database for later medical review (the user would not see it). An asthma specialist could later review and edit these draft answers and submit a final vetted answer for the FAQ database, thus reducing unanswerable questions over time. In this way, generative AI could be used as a workflow efficiency tool, if not a conversational agent in its own right.

We make a final point suggesting specialised digital health chatbots are still needed, even in an age of general-purpose AI chatbots, which relate to the specialised nature of health communication. General-purpose chatbots respond in set ways – often by providing bullet lists of informational points in a friendly tone. However, they cannot currently guide a patient through a conversational journey. In our conversations with specialists, the physicians described patient sessions as often involving open-ended questions and incremental guidance, for example, to support patients in discovering possible asthma triggers and in discerning how open they might be to changing behaviours (e.g. smoking) and then customising their responses to be as relevant as possible for that patient. LLMs are currently incapable of this type of multi-step, autonomy-supportive conversation. In this study, our chatbot included a scripted conversational module roughly modelled after a typical doctor–patient conversation about asthma triggers. It included questions like ‘Do you have a pet?’ And if the answer was no, then ‘When you go to friends’ houses that have pets, do you find your breathing gets affected?’ This conversational path then led to sensitive proposals for possible strategies, for example:

‘*I wouldn’t tell anyone to give away their pet, but you might consider keeping them out of the bedroom to see if your breathing improves. Do you think you might consider trying this strategy?*’

The difficulty in crafting supportive exploratory medical conversations with many branches – of the kind physicians and nurses have every day – is that it is resource intensive to map out the many pathways with sufficient richness. However, we believe it is potentially the greatest opportunity space for health chatbots, and we may find that LLMs can assist in the development process if not directly in patient interaction.

In summary, while general-purpose AI Chatbots can provide summaries of information that are often accurate, it remains up to specialised health chatbots to provide genuine safety, accuracy and well-crafted health conversations that can support patients in a process of reflection or behaviour change.

Of course, the landscape is changing so rapidly that decisions like these must be reassessed continually as future innovations and risks come to light. Moreover, despite the risks, people already use services like ChatGPT, Claude and Bard to answer their health questions.^41^ This is why any future research-based health chatbot, whether specialised for asthma or any other condition, must provide a higher standard of accuracy, safety and medical endorsement to provide distinctive value. Significant resources will also be needed to develop a sophisticated, broadly scoped conversational space.

The next steps for Brisa should involve meeting the demands of both accuracy and a fluent user experience. Improving fluency will address the causes of user dissatisfaction and likely resolve areas of low engagement. Fleshing out the conversation tree such that it incorporates more asthma triggers and more conversational pathways generally is one safe (if resource-intensive) approach to meeting these demands. In addition**,** to address the inevitable limitations of a pre-scripted dialogue tree, future research could explore the strengths and weaknesses of controlled GAI methods, such as retrieval augmented generation (RAG),^42^ that are designed to produce safer, contextually appropriate responses within set boundaries. Some combination of pre-scripted vetted responses for high-risk topics (such as asthma attack management) combined with generative but safeguarded responses may provide an effective pathway.

Finally, future research will also need to demonstrate medical efficacy. A feasibility study, while an essential and efficient first step towards developing an effective health intervention, is insufficient for determining statistically significant changes to health outcomes. Future research should implement the findings of this study to create a final version of the chatbot and test its uptake and impact on asthma health measures longitudinally across a larger representative population and of asthma patients as part of an RCT. An RCT can determine measurable changes to health literacy, self-management behaviours and critically, asthma health outcomes (e.g. improvements to asthma control measures, reduction in asthma attacks and reduced hospitalisations).

## Limitations

We note several limitations in this work. Firstly, while the depth of our entry and exit questionnaires contributed to our results, the number of questions (around 40 per survey) may have limited the response rate and the number of people willing to provide detailed feedback after using the chatbot (just 69). The financial compensation offered was designed to mitigate this burden. However, prior research has suggested that offering rewards for participation has little bearing on the response rate in online surveys.^43^ Additionally, the method of recruiting participants through social media sites may have narrowed our potential participant base, requiring an active account for visibility of the recruitment advertisement.

While the use of predefined conversational paths and a decision-tree structure in chatbot design ensures safety by allowing precise control over the dialogue presented to the user, this approach also places hard limits on the scope of conversational topics and on the chatbot's ability to provide varied and seamlessly natural conversational interaction that users value.^5^

In addition, for the feasibility study, we limited Brisa's knowledge to only four asthma trigger types, thus overlooking other types of common triggers, such as smoking, stress and exercise, among others. While this limitation was necessary given the project's resource constraints, expanding knowledge to other triggers would improve user experience and might be ratified through GAI. Additionally, integrations utilising external APIs and geolocation data, for example, could provide real-time awareness of local pollen counts and related risks.

## Conclusions

The feasibility study described herein provides evidence of a chatbot's value and potential efficacy for addressing aspects of the basic care gap in asthma, thereby improving asthma outcomes. However, it also highlights rising expectations around chatbot user experience, which we propose has resulted from the introduction of GAI chatbots such as ChatGPT. As such, health chatbots must now meet higher conversational flexibility, breadth and fluency standards to be effective. Because current LLM-based services still produce hallucinations and inaccuracies – even when fine-tuned or constrained – integrating these without expert oversight cannot currently solve the fluency problem while ‘first doing no harm’. Therefore, health chatbot development teams must devise creative solutions that will likely require significant resourcing to develop rich but safety-assured conversational spaces.

## Supplemental Material

sj-docx-1-dhj-10.1177_20552076241258276 - Supplemental material for A text-based conversational agent for asthma support: Mixed-methods feasibility study Supplemental material, sj-docx-1-dhj-10.1177_20552076241258276 for A text-based conversational agent for asthma support: Mixed-methods feasibility study by Darren Cook, Dorian Peters, Laura Moradbakhti, Ting Su, Marco Da Re, Bjorn W. Schuller, Jennifer Quint, Ernie Wong and Rafael A. Calvo in DIGITAL HEALTH

sj-pdf-2-dhj-10.1177_20552076241258276 - Supplemental material for A text-based conversational agent for asthma support: Mixed-methods feasibility study Supplemental material, sj-pdf-2-dhj-10.1177_20552076241258276 for A text-based conversational agent for asthma support: Mixed-methods feasibility study by Darren Cook, Dorian Peters, Laura Moradbakhti, Ting Su, Marco Da Re, Bjorn W. Schuller, Jennifer Quint, Ernie Wong and Rafael A. Calvo in DIGITAL HEALTH

sj-docx-3-dhj-10.1177_20552076241258276 - Supplemental material for A text-based conversational agent for asthma support: Mixed-methods feasibility study Supplemental material, sj-docx-3-dhj-10.1177_20552076241258276 for A text-based conversational agent for asthma support: Mixed-methods feasibility study by Darren Cook, Dorian Peters, Laura Moradbakhti, Ting Su, Marco Da Re, Bjorn W. Schuller, Jennifer Quint, Ernie Wong and Rafael A. Calvo in DIGITAL HEALTH

sj-docx-4-dhj-10.1177_20552076241258276 - Supplemental material for A text-based conversational agent for asthma support: Mixed-methods feasibility study Supplemental material, sj-docx-4-dhj-10.1177_20552076241258276 for A text-based conversational agent for asthma support: Mixed-methods feasibility study by Darren Cook, Dorian Peters, Laura Moradbakhti, Ting Su, Marco Da Re, Bjorn W. Schuller, Jennifer Quint, Ernie Wong and Rafael A. Calvo in DIGITAL HEALTH

## Notes

a.
https://www.prolific.com/

b. A score of 0.9 or higher was deemed high risk, following consultation with medical experts.

c. Notifications via WhatsApp were only sent during Wave 3 of the feasibility study. All other notifications were sent via users’ email addresses when completing the entry questionnaire.

d. An active user is an individual who interacted with Brisa at least once within 28 days.

e. Bonferroni correction applied.

f. Due to a processing error, user satisfaction data was only collected for Waves 2 and 3.

g. The denominator is the 69 users who completed this portion of the exit survey.

h. NPS is calculated by subtracting the percentage of detractors (scores between 0 and 6) from the percentage of promotors (scores between 9 and 10). NPS ranges between –100 and 100, with a high positive NPS indicating high user satisfaction.
