# Supplementary material for: A text-based conversational agent for asthma support: Mixed-methods feasibility study
Source: Digit Health. 2024 Jun 17;10:20552076241258276. doi: 10.1177/20552076241258276 (PMC11185032; doi:10.1177/20552076241258276)
Supplement: sj-docx-1-dhj-10.1177_20552076241258276 - Supplemental material for A text-based conversational agent for asthma support: Mixed-methods feasibility study [file sj-docx-1-dhj-10.1177_20552076241258276.docx]

# Appendices

## Appendix A: Examples of social media advertisement.


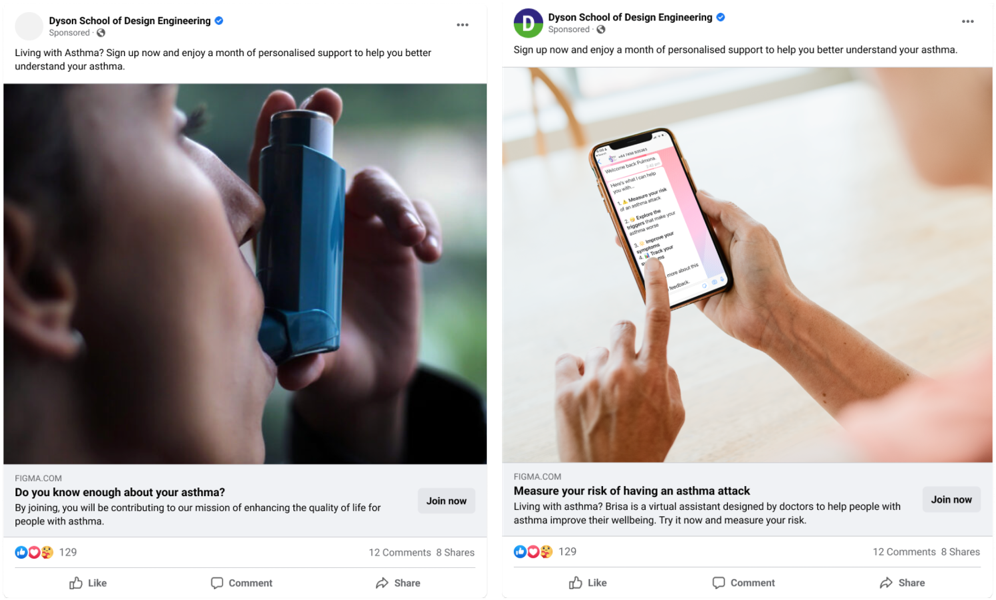


## Appendix B: Complete list of questions asked by the user during FAQ.

The following questions were generated via our team of asthma specialists or asked by users during conversations with Brisa.

| Question |
| --- |
| What are the symptoms of asthma? |
| How is asthma diagnosed? |
| How can I better manage asthma? |
| Will I ever stop having asthma? |
| Is asthma curable? |
| Will I die from having asthma? |
| What causes asthma? |
| How does my inhaler help with asthma? |
| What are the treatments for asthma? |
| Is asthma treatment free? |
| Why do I have asthma? |
| What does poor asthma control look like? |
| When should I monitor my peak flow? |
| My partner says that I cough at night, but this is not waking me up. Why is this considered poor asthma? |
| How can I improve my asthma? |
| What is happening inside my lungs when my asthma is not well controlled? |
| Where can I get support for my asthma? |
| Which websites do you recommend for information on asthma? |
| Why should I be seen by a healthcare provider after I have had an asthma attack? |
| How do my inhalers work? |
| What is the difference between my inhalers? |
| Aren't spacers for kids? |
| Does it matter what time I take my inhalers? |
| Why do I need to rinse my mouth after using ICS? |
| Will inhaled steroids make me put on weight? |
| Why do I need several inhalers? |
| Will I be using inhalers for the rest of my life? |
| Why do I need a flu jab? |
| Will it make any difference if I lose weight? |
| Why have my symptoms returned after not having any for so long? |
| Are inhalers safe if I'm pregnant? |
| Does weather affect my asthma? |
| What is the weather like? |
| Is asthma hereditary? |
| How to cope with hot weather? |
| What will happen if I accidently eat something I’m allergic to? |
| Asthma in workplace? |
| When to go to A&E? |
| Can you tell me about ventolin? |
| Fostair is giving me cramps? |
| What does atopic mean? |
| How long does a MDI last once opened meaning expired rather than doses? |
| Is there any indoor plants that are good for my asthma? |
| Is carpet bad for asthma? |
| How does hay fever affect my asthma? |
| How to vacuum effectively to prevent asthma? |
| How to stop coughing? |
| Can you tell me about fostair nexthaler? |
| How often is normal to have an asthma attack? |
| What's brittle asthma? |
| Is it ok to take montelukast during the day? |
| Why do I cough up so much phlegm? |
| Track my asthma? |
| How do I know if I have a chest infection? |

## Appendix C: Example of a PDF report


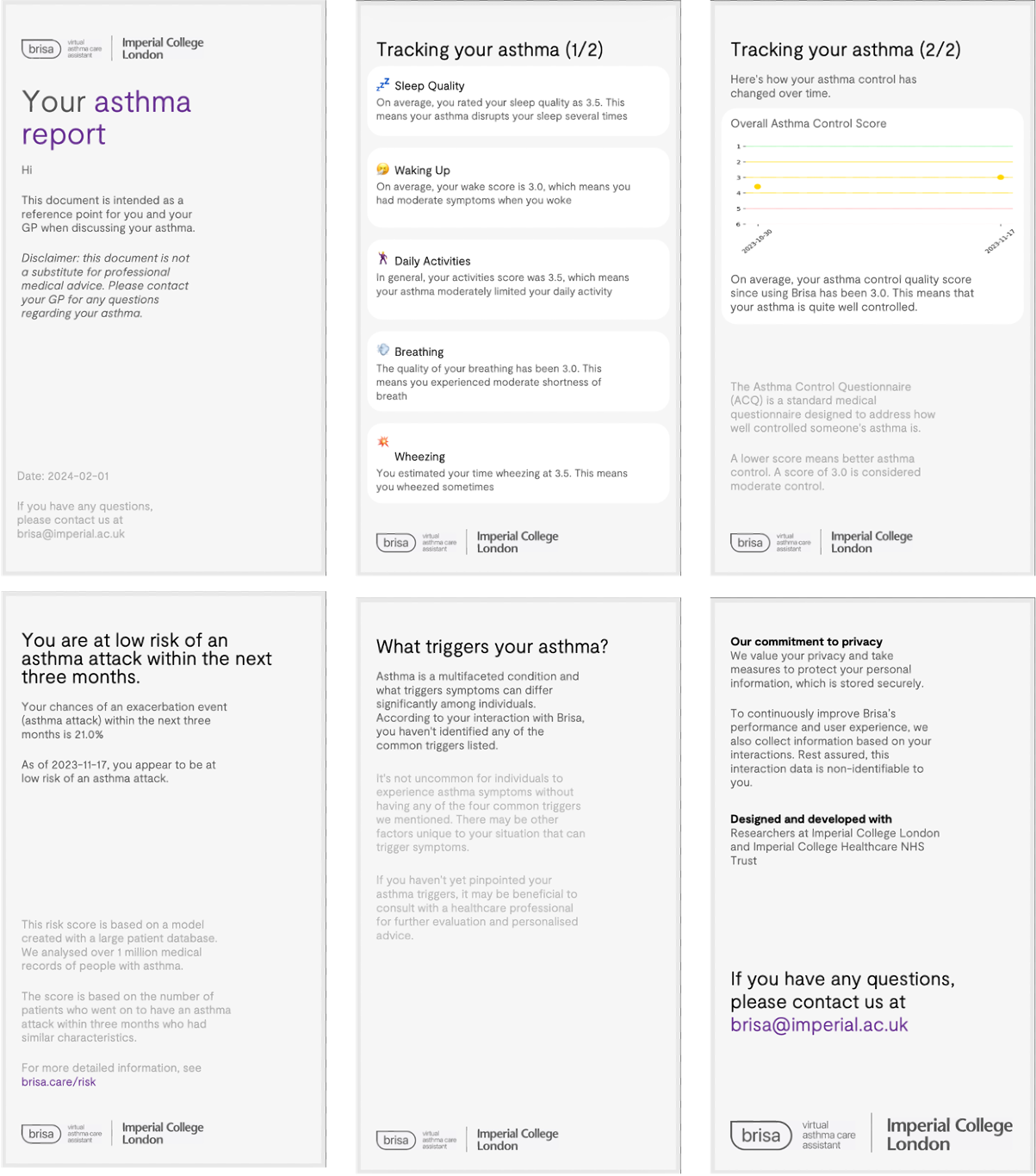


## Appendix D: Comparing standardised and generated text on asthma attacks.

We asked three LLM-based AI Chatbots, “What should I do if I'm having an asthma attack?” and then compared responses to the highly refined standard national advice text.

**NHS and Asthma and Lung UK:**
The standard text used on the NHS and Asthma and Lung UK websites is a simple and clear 5-step response. It includes an important caveat for patients using a specific medication regime (“MART”) that links to different advice. Specifically, at the time of testing, the Asthma and Lung UK text under the heading “What to do in an asthma attack” was:

If you think you're having an asthma attack, you should:

1. Sit up straight – try to keep calm.
2. Take one puff of your reliever inhaler (usually blue) every 30 to 60 seconds, up to 10 puffs.
3. If you feel worse at any point or do not feel better after 10 puffs, **call 999 for an ambulance**.
4. If the ambulance has not arrived after 10 minutes and your symptoms are not improving, repeat step 2.
5. If your symptoms are no better after repeating step 2, and the ambulance has still not arrived, **contact 999 again immediately**.

**Important**: this asthma attack advice does not apply to you if you use a MART inhaler.

**Claude by Anthropic**

‘Claude’, the freely available AI Chatbot created by Anthropic^[[1]](#footnote-1)^, generated a longer, less clear, slightly repetitive bullet list of advice points, and critically, it had no instructions for people using the MART inhaler. Claude’s response was:

If you are having an asthma attack, it's important to follow these steps:

- Try to keep calm and sit in an upright position to allow your lungs to expand.
- Take your quick-relief inhaler (usually albuterol) as prescribed. You may need to take 2-4 puffs every 20 minutes for 1 hour.
- If you do not have quick relief medication or if symptoms persist, call emergency services right away. Asthma attacks can become life-threatening.
- Use your peak flow meter to measure your lung function if one is available. Compare the reading to your personal best. Readings below 50% warrant emergency care.
- Take slow, deep breaths. Try pursed lip breathing - breathe in through your nose and breathe out slowly through pursed lips.
- Clear any mucus by sipping warm water or tea, but do not lie down. Stay upright.
- If approved by your doctor, take oral steroids at the earliest sign of an attack to reduce inflammation.
- Avoid triggers that may worsen symptoms - smoke, cold air, allergens, exercise, etc.
- Monitor your condition carefully. Seek medical help or call 911 if you have no improvement after initial treatment, you're struggling for breath, your lips are blue, or you're unable to speak in full sentences.

The key is remaining calm, following your asthma action plan, and monitoring your symptoms closely. Seek immediate medical attention if you are not improving or symptoms are worsening rapidly.”

**ChatGPT Plus by OpenAI**
The paid version of ChatGPT used GPT-4 at the time of use. ChatGPT was the only chatbot of the three tested to caution the user that it was not a medical expert, but it proceeded to give advice anyway. Like Claude, it also produced longer and less specific instructions than the standard approved text (e.g., didn’t include the required number of puffs per timeframe). It also excluded MART users. The response generated by ChatGPT was:

I'm not a doctor, but I can provide some general advice on what to do during an asthma attack. It's important to follow your asthma action plan if you have one. If you are experiencing an asthma attack and it's severe or your symptoms are worsening, seek emergency medical attention immediately. Here are some steps you can follow:

1. **Stay Calm**: Try to remain as calm as possible. Panic can worsen symptoms.
2. **Sit Upright**: Do not lie down. Sitting upright can help you breathe more easily.
3. **Use Your Rescue Inhaler**: Use your quick-relief (rescue) inhaler as directed. This is usually a blue inhaler that contains a bronchodilator like albuterol.
4. **Breathe Slowly and Deeply**: This helps the medicine get deep into your lungs and calms your breathing. Some people find it helpful to follow a specific breathing technique.
5. **Follow Your Asthma Action Plan**: If you have an asthma action plan prescribed by your doctor, follow its instructions.
6. **Seek Emergency Care if Needed**: If symptoms do not improve after using the rescue inhaler, if they return quickly, or if you're unsure, seek emergency medical care. Symptoms of a severe asthma attack that require immediate medical attention include trouble walking or talking due to shortness of breath, lips or fingernails turning blue, or no improvement after using the rescue inhaler.

Remember, it's essential to manage your asthma with regular check-ups with your healthcare provider and by following your treatment plan, including taking preventive medication if prescribed.

If you're unsure about what to do or if you're having severe symptoms, seek immediate medical attention.

**CoPilot by Microsoft**

‘CoPilot” is an AI chatbot provided for free by Microsoft via its ‘Edge’ Browser. We left the precision setting at the default value “balanced”. This chatbot produced arguably the best result as it reproduced the NHS text nearly verbatim with citations to the source and provided links to reliable sources, including the NHS and Red Cross websites, at the end. However, it *still* omitted the caveat and instructions for MART users. Furthermore, certain phrases in the text were underlined as links. These links triggered advertisements for asthma travel insurance on hover (see Appendix Figure 1). The CoPilot response was:

If you are having an asthma attack, it is important to act quickly. Here are the steps you should follow:

1. **Sit up straight** and try to remain calm.
2. Take one puff of your **reliever inhaler** (usually blue) every 30 to 60 seconds, up to 10 puffs.
3. If you feel worse at any point, or you do not feel better after 10 puffs, **call** **999** for an ambulance.
4. If the ambulance has not arrived after 10 minutes and your symptoms are not improving, repeat step 2.
5. If your symptoms are no better after repeating step 2, and the ambulance has still not arrived, **contact 999 again immediately**.

It is important to never be frightened of calling for help in an emergency. Try to take the details of your medicines (or your personal asthma action plan) with you to hospital if possible. After an asthma attack, you should see a GP or asthma nurse within 48 hours of leaving the hospital, or ideally on the same day if you did not need hospital treatment 123.

If you are experiencing worsening symptoms despite using a quick-relief (rescue) inhaler, or if you have low peak expiratory flow (PEF) readings, you may be having an asthma attack. Other signs and symptoms of an asthma attack include severe shortness of breath, chest tightness or pain, and coughing or wheezing ^1^. If you are on the right asthma treatment, your chance of having an attack is greatly reduced. Visit a doctor or asthma nurse at least once a year for a check-up and to discuss your treatment^2^.

I hope this information helps. Let me know if you have any other questions.

**Learn more.**

^1^ bing.com

^2^ nhs.uk

^3^ Redcross.org.uk"


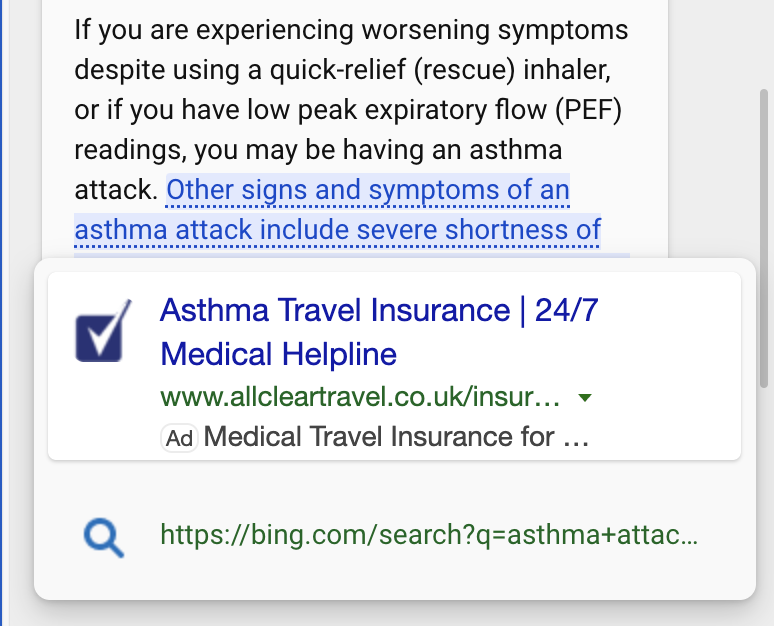

*Appendix Figure 1. Screenshot of pop-up advertising embedded in the asthma attack response provided by Microsoft’s CoPilot.*

1. https://www.anthropic.com [↑](#footnote-ref-1)
