# Supplementary material for: A text-based conversational agent for asthma support: Mixed-methods feasibility study
Source: Digit Health. 2024 Jun 17;10:20552076241258276. doi: 10.1177/20552076241258276 (PMC11185032; doi:10.1177/20552076241258276)
Supplement: sj-docx-3-dhj-10.1177_20552076241258276 - Supplemental material for A text-based conversational agent for asthma support: Mixed-methods feasibility study [file sj-docx-3-dhj-10.1177_20552076241258276.docx]

Brisa Wave 3 Screening and Pre-Questionnaire

Start of Block: End of Wave 3 Notice

Start of Block: Landing Section

Intro Assessing the Design of Conversational Agents for Asthma Support
 Thank you for showing interest in trying out our **virtual asthma assistant**. This is a **research study** that allows you to try Brisa and help us assess the design of this virtual assistant. Brisa is an asthma chatbot created by a research team at Imperial College London. We have worked with asthma doctors and patients to deliver a chatbot that can help you improve your asthma.   We have now finished recruiting new users to test out our chatbot. Please see our website for further information and future opportunities to try out Brisa. If you would like to reach out to us, please email brisa@imperial.ac.uk

 Thank you for your interest,

 **Wellbeing Technologies Lab
 Dyson School of Design Engineering
 Imperial College London**

| Page Break |  |
| --- | --- |

Q106 Here’s what this research study involves:
 📝 Fill out a **starting questionnaire** (5 mins). 💬 Get access to the chatbot for **4 weeks**. 📝After 4 weeks, fill out an **exit questionnaire** (5 mins). 🎁 Receive **£10,** a thank you gift for your help.
 Before you decide if to be part of this, it's important for you to understand why the research is being done and what it will involve. Please read the information about the study in the participant information sheet.
Click "Next" to move forward.

| Page Break |  |
| --- | --- |

CAPTCHA Before proceeding to the survey, please complete the captcha below.

End of Block: Landing Section

Start of Block: UK Screener

Screen for UK-based Are you based in the UK?
 Note: You will need a UK-based phone number if you intend to use Brisa via WhatsApp

- Yes
- No

End of Block: UK Screener

Start of Block: Previous Access Screener

For previous users Have you volunteered to test a previous version of Brisa before?
 Note: Previous users will be unable to participate in this study for a second time.

- Yes
- No

End of Block: Previous Access Screener

Start of Block: Consent

Q95 **Consent to participate in the study "Improving asthma care through personalised risk assessment and support from a conversational agent"**

Consent 1 I confirm that I have read and understand the Participant Information Sheet version 1.5 dated 06/10/23 for the above study and have had the opportunity to ask questions which have been answered fully.

- Yes
- No

Consent 2 I understand that my participation is voluntary, and I am free to withdraw at any time, without giving any reason and without my legal rights nor treatment/ healthcare being affected.

- Yes
- No

Consent 3 I give consent for information collected about me to be used to support other research or in the development of a new test, medication, medical device or treatment by an academic institution or commercial company in the future, including those outside of the United Kingdom (which Imperial has ensured will keep this information secure).

- Yes
- No

Consent 4 I understand that data collected from me are a gift donated to Imperial College and that I will not personally benefit financially if this research leads to an invention and/or the successful development of a new test, medication treatment, product or service.

- Yes
- No

Consent 5 I give consent to being contacted about the possibility to take part in other research studies.

- Yes
- No
- Click to write Choice 3

Consent 6 I give consent to submit a short voice recording to the chatbot as one of the steps to assess my asthma symptoms.

- Yes
- No

Consent 7 I consent to take part in the current study called: Improving asthma care through personalised risk assessment and support from a conversational agent.

- Yes
- No

Full Name Please enter your full name:

________________________________________________________________

End of Block: Consent

Start of Block: Consent given

Survey Questions Thank you for agreeing to participate in this research study. Your help is essential in advancing asthma research.
   **Welcome!** We would like to introduce you to *Brisa*, a chatbot that can assist you in managing your asthma symptoms.  
 If you are still interested in trying out *Brisa*, please answer the following questions first to help us better understand your asthma journey.

| Page Break |  |
| --- | --- |

Q41 Demographics

| 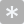 |
| --- |

Age  How old are you?

________________________________________________________________

Gender What is your gender?

- Male
- Female
- Non-binary
- Prefer not to say

Minority Do you identify with any particular ethnic or cultural background, including but not limited to Black, Indigenous, Asian, or mixed ethnicity?

- Yes
- No

Education What is the highest level of education you have completed?

- Did not complete secondary school
- Secondary school
- College
- Undergraduate degree (University)
- Postgraduate degree
- Other (specify) __________________________________________________

| Page Break |  |
| --- | --- |

Q42 Trust in healthcare

Trust in Healthcare How much do you agree or disagree with the following statement: "I trust the UK healthcare system"

- Strongly agree
- Agree
- Neutral
- Disagree
- Strongly disagree

| Page Break |  |
| --- | --- |

Q43 Technology experience

Confidence in Tech Overall, how confident do you feel using computers, smartphones or other digital devices to do the things you need to do online?

- Highly confident
- Confident
- Neither
- Not confident
- Not confident at all

Messaging Apps Usage How often do you use messaging apps on a smartphone (e.g. WhatsApp, Messenger, SnapChat etc.)?

- Several times a day
- Once a day
- A few times per week
- Less than once a week
- Never

Whats App How often do you use WhatsApp specifically?

- Several times a day
- Once a day
- A few times per week
- Less than once a week
- Never

Mobile App Asthma Have you ever used a mobile app to help you with asthma (for example, to get information about asthma or to track your symptoms)?

- Yes
- No

| Page Break |  |
| --- | --- |

Asthma Severity Qs **The following questions help us to better understand your asthma journey.**
 Please answer the following questions on a 7-point scale.

Asthma Night On average, during the past week, how often were you woken by your asthma during the night?

- 1 - Never
- 2 - Hardly ever
- 3 - A few times
- 4 - Several times
- 5- Many times
- 6 - A great amount of times
- 7 - Unable to sleep because of asthma

Asthma Morning On average, during the past week, how bad were your asthma symptoms when you woke up in the morning?

- 1 - No symptoms
- 2 - Very mild
- 3 - Mild
- 4 - Moderate
- 5 - Quite severe
- 6 - Severe
- 7 - Very severe

Asthma Activities In general, during the past week, how limited were you in your activities because of your asthma?

- 1 - Not limited
- 2 - Very slightly limited
- 3 - Slightly limited
- 4 - Moderately limited
- 5 - Very limited
- 6 - Extremely limited
- 7 - Totally limited

Shortness of Breath In general, during the past week, how much shortness of breath did you experience because of your asthma?

- 1 - None
- 2 - Very little
- 3 - A little
- 4 - A moderate amount
- 5 - Quite a lot
- 6 - A great deal
- 7 - A very great deal

Wheeze In general, during the past week, how much of the time did you wheeze?

- 1 - Not at all
- 2 - Hardly any of the time
- 3 - A little of the time
- 4 - A moderate amount of the time
- 5 - A lot of the time
- 6 - Most of the time
- 7 - All of the time

| Page Break |  |
| --- | --- |

Q78 Pre-Usage Questionnaire Please answer the next two questions on a 7-point scale from strongly disagree to strongly agree. These questions refer to the Asthma Chatbot Brisa which you will be able to try out in the next step.

Effective Use I feel confident that I'll be able to use the asthma chatbot *Brisa* effectively

- 1 (strongly disagree)
- 2
- 3
- 4
- 5
- 6
- 7 (strongly agree)

Easy to Use The asthma chatbot *Brisa* will be easy for me to use

- 1 (strongly disagree)
- 2
- 3
- 4
- 5
- 6
- 7 (strongly agree)

| Page Break |  |
| --- | --- |

Brisa Usage And finally, how would you like to interact with *Brisa*?

- WhatsApp
- Web browser

End of Block: Consent given

Start of Block: Browser option selected

| 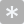 |
| --- |

Q10 To interact with *Brisa* via a web browser, you'll need to use your email to get started. Please enter your email below to receive a link.

________________________________________________________________

| 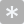 |
| --- |

Q103 Finally, please provide the UK mobile phone number (beginning 07 or +44) to receive your £10 voucher at the end of your participation.

________________________________________________________________

End of Block: Browser option selected

Start of Block: WhatsApp option selected

| 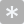 |
| --- |

Q86 To interact with *Brisa* via WhatsApp, you'll need to provide an email so we can send you a personalised link.  **Please enter your email below to receive a link.**

________________________________________________________________

| 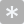 |
| --- |

Q94 Finally, please provide the **UK mobile phone number** (beginning 07 or +44) you intend to use to access Brisa.

________________________________________________________________

End of Block: WhatsApp option selected
