# Supplementary material for: A text-based conversational agent for asthma support: Mixed-methods feasibility study
Source: Digit Health. 2024 Jun 17;10:20552076241258276. doi: 10.1177/20552076241258276 (PMC11185032; doi:10.1177/20552076241258276)
Supplement: sj-docx-4-dhj-10.1177_20552076241258276 - Supplemental material for A text-based conversational agent for asthma support: Mixed-methods feasibility study [file sj-docx-4-dhj-10.1177_20552076241258276.docx]

Post-Questionnaire Brisa Wave 3

Start of Block: Intro

Thank you for testing the **Asthma Chatbot**!

The following questions are about your experience using the chatbot. Your honest feedback will help make it better.

| 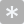 |
| --- |

To get started, please enter the email address you used to sign-up for Brisa.

________________________________________________________________

End of Block: Intro

Start of Block: Heuristics

The **language** and **tone of voice** used by the chatbot was appropriate.

- 5 Strongly agree
- 4 Agree
- 3 Neutral
- 2 Disagree
- 1 Strongly disagree

The conversation felt **natural**.

- 5 Strongly agree
- 4 Agree
- 3 Neutral
- 2 Disagree
- 1 Strongly disagree

The chatbot responded in a **consistent** manner.

- 5 Strongly agree
- 4 Agree
- 3 Neutral
- 2 Disagree
- 1 Strongly disagree

The chatbot gave me **options** that were easy to follow.

- 5 Strongly agree
- 4 Agree
- 3 Neutral
- 2 Disagree
- 1 Strongly disagree

It was **easy to understand** what I could do with the chatbot and how I should do it.

- 5 Strongly agree
- 4 Agree
- 3 Neutral
- 2 Disagree
- 1 Strongly disagree

I had **enough control** over the conversation.

- 5 Strongly agree
- 4 Agree
- 3 Neutral
- 2 Disagree
- 1 Strongly disagree

The conversation had **no** **bugs** or abrupt endings.

- 5 Strongly agree
- 4 Agree
- 3 Neutral
- 2 Disagree
- 1 Strongly disagree

it was **easy to get back on track** if I made the wrong choice or typed the wrong thing.

- 5 Strongly agree
- 4 Agree
- 3 Neutral
- 2 Disagree
- 1 Strongly disagree

It was easy to **return to the conversation** at a later time if I was interrupted.

- 5 Strongly agree
- 4 Agree
- 3 Neutral
- 2 Disagree
- 1 Strongly disagree

End of Block: Heuristics

Start of Block: Heuristics 2

The chatbot's responses were **helpful**

- 5 Strongly agree
- 4 Agree
- 3 Neutral
- 2 Disagree
- 1 Strongly disagree

The chatbot **remembered** things that I told it

- 5 Strongly agree
- 4 Agree
- 3 Neutral
- 2 Disagree
- 1 Strongly disagree

The chatbot responded in a way that was **personalised** to my situation

- 5 Strongly agree
- 4 Agree
- 3 Neutral
- 2 Disagree
- 1 Strongly disagree

I felt like I could trust the **accuracy** of the chatbot's responses

- 5 Strongly agree
- 4 Agree
- 3 Neutral
- 2 Disagree
- 1 Strongly disagree

I trust that my conversations with the chatbot were **private** **and secure**.

- 5 Strongly agree
- 4 Agree
- 3 Neutral
- 2 Disagree
- 1 Strongly disagree

I felt like I could **communicate in the way I wanted** to with the chatbot.

- 5 Strongly agree
- 4 Agree
- 3 Neutral
- 2 Disagree
- 1 Strongly disagree

The chatbot helped me feel more **confident** I can manage my asthma.

- 5 Strongly agree
- 4 Agree
- 3 Neutral
- 2 Disagree
- 1 Strongly disagree

When I used the Asthma Chatbot, I felt **less alone** in managing my asthma.

- 5 Strongly agree
- 4 Agree
- 3 Neutral
- 2 Disagree
- 1 Strongly disagree

When I used the Asthma Chatbot, I felt like the **interaction** went both ways.

- 5 Strongly agree
- 4 Agree
- 3 Neutral
- 2 Disagree
- 1 Strongly disagree

End of Block: Heuristics 2

Start of Block: Q4TE, info quantity and pace

In general, how was the **amount of information** the chatbot provided in its responses? 

- Not enough
- The right amount
- Too much

In general, how was the **pace** of the chatbot's responses?

- Too slow (responses took too long)
- About right
- Too fast (responses went by too quickly)

I enjoyed using the chatbot.

- 5 Strongly agree
- 4 Agree
- 3 Neutral
- 2 Disagree
- 1 Strongly disagree

Using the chatbot was beneficial to me.

- 5 Strongly agree
- 4 Agree
- 3 Neutral
- 2 Disagree
- 1 Strongly disagree

After using the chatbot, I know more about asthma in general, or about *my* asthma, than I did before.

- 5 Strongly agree
- 4 Agree
- 3 Neutral
- 2 Disagree
- 1 Strongly disagree

I learned new things from using the chatbot.

- 5 Strongly agree
- 4 Agree
- 3 Neutral
- 2 Disagree
- 1 Strongly disagree

End of Block: Q4TE, info quantity and pace

Start of Block: Images

The next question is about the images used by the chatbot.

I liked the images used by the chatbot and found them useful

- 5 Strongly agree
- 4 Agree
- 3 Neutral
- 2 Disagree
- 1 Strongly disagree

Please share some reasons for your answer:

________________________________________________________________

________________________________________________________________

________________________________________________________________

________________________________________________________________

________________________________________________________________

End of Block: Images

Start of Block: Risk assessment

Did you use the chatbot to measure your ⚠️ risk of having an asthma attack?

- Yes
- No

Was your risk 🔵 low, 🟠 medium or 🔴 high?

- Low
- Medium
- High
- Unsure

How useful did you find the risk feature?

- Not useful
- Only slightly useful
- Somewhat useful
- Useful
- Very useful

Please share any reasons for your answer

________________________________________________________________

________________________________________________________________

________________________________________________________________

________________________________________________________________

________________________________________________________________

End of Block: Risk assessment

Start of Block: Triggers Dialog

Did you use the chatbot to discuss asthma **triggers** 🤧 or **strategies** 🔆 to manage triggers?

- Yes
- No

How useful did you find the conversation about triggers and strategies?

- Not useful
- Only slightly useful
- Somewhat useful
- Useful
- Very useful

Please share any reasons for your answer

________________________________________________________________

________________________________________________________________

________________________________________________________________

________________________________________________________________

________________________________________________________________

End of Block: Triggers Dialog

Start of Block: AMA dialog

Did you use the '💬 Frequently Asked Question’ feature that allows you to ask the chatbot an open question?

- Yes
- No

How useful did you find the option to ask any question you wanted?

- Not useful
- Only slightly useful
- Somewhat useful
- useful
- Very useful

Please share any reasons for your answer

________________________________________________________________

________________________________________________________________

________________________________________________________________

________________________________________________________________

________________________________________________________________

End of Block: AMA dialog

Start of Block: Tracking dialog

Did you use the '📊 Track my asthma’ feature to track your level of asthma control?

- Yes
- No

How useful did you find the option to track your asthma over time?

- Not useful
- Only slightly useful
- Somewhat useful
- useful
- Very useful

Please share any reasons for your answer

________________________________________________________________

________________________________________________________________

________________________________________________________________

________________________________________________________________

________________________________________________________________

End of Block: Tracking dialog

Start of Block: ACQ

Almost done! The next 5 questions are about your asthma.

On average, during the past week, how often were you woken by your asthma during the night?

- 1 - Never
- 2 - Hardly ever
- 3 - A few times
- 4 - Several times
- 5- Many times
- 6 - A great amount of times
- 7 - Unable to sleep because of asthma

On average, during the past week, how bad were your asthma symptoms when you woke up in the morning?

- 1 - No symptoms
- 2 - Very mild
- 3 - Mild
- 4 - Moderate
- 5 - Quite severe
- 6 - Severe
- 7 - Very severe

In general, during the past week, how limited were you in your activities because of your asthma?

- 1 - Not limited
- 2 - Very slightly limited
- 3 - Slightly limited
- 4 - Moderately limited
- 5 - Very limited
- 6 - Extremely limited
- 7 - Totally limited

In general, during the past week, how much shortness of breath did you experience because of your asthma?

- 1 - None
- 2 - Very little
- 3 - A little
- 4 - A moderate amount
- 5 - Quite a lot
- 6 - A great deal
- 7 - A very great deal

In general, during the past week, how much of the time did you wheeze? Type the correct number...

- 1 - Not at all
- 2 - Hardly any of the time
- 3 - A little of the time
- 4 - A moderate amount of the time
- 5 - A lot of the time
- 6 - Most of the time
- 7 - All of the time

End of Block: ACQ

Start of Block: Notifications

Did you receive notifications from Brisa, either as an email or via WhatsApp?

- Yes
- No

End of Block: Notifications

Start of Block: Notification Efficacy

Did you find the notifications helpful?

- Yes
- No

What would have made notifications more useful?

________________________________________________________________

End of Block: Notification Efficacy

Start of Block: Net Promoter Score

On a scale of 0 to 10, how likely would you be to recommend the Asthma Chatbot to a friend or colleague with asthma?

- 0
- 1
- 2
- 3
- 4
- 5
- 6
- 7
- 8
- 9
- 10

What did you like best about the chatbot?

________________________________________________________________

What did you like least?

________________________________________________________________

How could the chatbot be improved?

________________________________________________________________

________________________________________________________________

________________________________________________________________

________________________________________________________________

________________________________________________________________

End of Block: Net Promoter Score

Start of Block: Voucher Code

Claim your £10 Amazon Voucher! Before you go... Please email us at brisa@imperial.ac.uk quoting code "**Brisa23**" to receive your voucher as a thank you for participating.

End of Block: Voucher Code
